# The potential utility of (2S,4R)-4-[18F] fluoroglutamine as a novel metabolic imaging marker for inflammation explored by rat models of arthritis and paw edema

**DOI:** 10.21203/rs.3.rs-4493375/v1

**Published:** 2024-06-20

**Authors:** Kim Min-Jeong, Hari K. Akula, Jocelyn Marden, Kaixuan Li, Bao Hu, Paul Vaska, Wenchao Qu

**Affiliations:** Stony Brook University Health Sciences Center School of Medicine: Stony Brook University Renaissance School of Medicine; Stony Brook University Health Sciences Center School of Medicine: Stony Brook University Renaissance School of Medicine; Stony Brook University Health Sciences Center School of Medicine: Stony Brook University Renaissance School of Medicine; Stony Brook University; Stony Brook University Health Sciences Center School of Medicine: Stony Brook University Renaissance School of Medicine; Stony Brook University Health Sciences Center School of Medicine: Stony Brook University Renaissance School of Medicine; Stony Brook University Health Sciences Center School of Medicine: Stony Brook University Renaissance School of Medicine

**Keywords:** (2S,4R)-4-[18F]fluoroglutamine, metabolic imaging, inflammation, glutamine

## Abstract

**Purpose:**

(*2S,4R*)-4-[^18^F]fluoroglutamine ([^18^F]FGln) is a promising metabolic imaging marker in cancer. Based on the fact that major inflammatory cells are heavily dependent on glutamine metabolism like cancer cells, we explored the potential utility of [^18^F]FGln as a metabolic imaging marker for inflammation in two rat models: carrageenan-induced paw edema (CIPE) and collagen-induced arthritis (CIA).

**Procedures:**

The CIPE model (n = 4) was generated by injecting 200 μL of 3% carrageenan solution into the left hind paw three hours before the PET. The CIA model (n = 4) was generated by injecting 200 μg of collagen emulsion subcutaneously at the tail base 3–4 weeks before the PET. A qualitative scoring system was used to assess the severity of paw inflammation. After a CT scan, 15.7 ± 4.9 MBq of [^18^F]FGln was injected via the tail vein, followed by a dynamic micro-PET scan for 90 minutes under anesthesia with isoflurane. The standard uptake value of [^18^F]FGln was measured by placing a volume of interest in each paw. The non-injected right hind paws of the CIPE model rats served as controls for both models. The paws with CIA were pathologically examined after PET.

**Results:**

In CIPE models, uptake in the injected paw was higher compared to the non-injected paw by 52–83%. In CIA models, uptake in the paws with severe inflammation was higher than the averaged controls by 54–173%, while that with mild and no inflammation was slightly higher (33%) and lower (−7%), respectively. Combined overall, the [^18^F]FGln uptake in CIA showed a significant positive correlation with inflammation severity (*r* = 0.88, *P* = 0.009). The pathological findings confirmed profound inflammation in CIA.

**Conclusions:**

[^18^F]FGln uptake was increased in both acute and chronic inflammation, and the uptake level was significantly correlated with the severity, suggesting its potential utility as a novel metabolic imaging marker for inflammation.

## Introduction

(*2S,4R*)-4-[^18^F] fluoroglutamine ([^18^F]FGln) has shown promise as a metabolic imaging marker using positron emission tomography (PET) technique in various types of cancer [1–4]. For instance, a recent study in human subjects suggested that [^18^F]FGln is more specific to the metabolic activities of cancer cells avid to glutamine compared to [^18^F]fluoro-D-glucose ([^18^F]FDG), which is currently the standard PET imaging marker to monitor cancer [5]. However, [^18^F]FGln has been rarely evaluated in non-cancerous disease conditions such as inflammation, despite the ample evidence of the heavy metabolic dependence of major inflammatory cells on glutamine as well. Specifically, glutaminolysis, a conversion process from glutamine into glutamate, aspartate, and alanine, is an essential metabolic step to provide energy sources for the proliferation of inflammatory cells and their reactions against external or internal pathogens [6]. Thus, [^18^F]FGln has great potential to be used to monitor inflammation in addition to cancer, and we aimed to explore the utility of the [^18^F]FGln as a novel metabolic imaging marker for inflammation in well-established rat models of acute and chronic inflammation.

Two distinct rat models were used in this study: the carrageenan-induced paw edema (CIPE) model for local and acute inflammation, and the collagen-induced arthritis (CIA) model for systemic and chronic inflammation. Carrageenan is a seaweed-derived sulfated polysaccharide that has been extensively used to induce inflammation in experimental animal models to study novel anti-inflammatory and analgesic drugs [7]. In the CIPE model, carrageenan injected into the rodents’ hind paws causes a classical innate immune response characterized by paw edema, neutrophil migration, and pain [8–9]. CIA is an experimental model of autoimmune arthritis and has been shown to exhibit similar histological, immunological, and clinical characteristics and genetic linkage to human rheumatoid arthritis (RA) [10–11]. This model has been widely used in studies on the pathogenesis of RA and the preclinical evaluation of novel therapeutics for RA [12]. The protocol for the generation of the CIA model is well-established with high reproducibility [13].

## Materials and Methods

### Radiosynthesis of [^18^F]FGln

**Figure 1:**
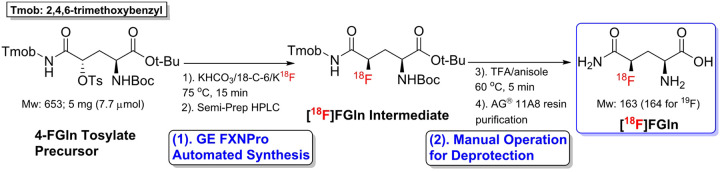
Synthesis of (*2S,4R*)-4-[^18^F] fluoroglutamine ([^18^F]FGln) for imaging paw inflammation: (1). Automated flnucleophilic [^18^F] fluorination; (2). Manual-operated acidic deprotection and purification.

[^18^F]FGln was prepared through a semi-automated synthesis process (Fig. 1) [14],[15]: 1) The ^18^F-labeled intermediate was prepared following an automated production protocol reported by Zhang et al. using GE^™^ TRACERlab FXNPro module; 2) The acidic deprotection and purification were conducted in a manual operation manner following the initial [^18^F]FGln synthesis report with minor modification. After the [^18^F]FGln intermediate was collected from semi-prep HPLC, it was first concentrated using the solid phase extraction (SPE) method. The trapped radioactivity was next eluted with EtOH (1 mL), and the solvent was removed by azeotropic drying of the intermediate solution under a gentle nitrogen stream. Once all solvent was removed, the acidic deprotection (trifluoroacetic acid, TFA) and the following ion-retardation resin removal of TFA provided the desired product [^18^F]FGln. The total production time ranged from 86 to 110 min. Starting with 9.1–15.2 GBq uorine-18 activity, 166–411 mBq amount of [^18^F]FGln was produced at the end of synthesis with the radiochemical purities of the final product range from 89–94%.

### Generation of animal models

The experimental protocol for the generation of the CIPE and CIA models and micro-PET scans was approved by the Institutional Animal Care and Use Committee at Stony Brook University (IACUC2022–00034).

The CIPE model (n = 4, Lewis female rats, Charles River Laboratories) was generated by injecting 200 μL of 3% carrageenan solution into the left hind paw subplantarly as previously described [16] under general anesthesia with isoflurane three hours before the PET scan. Carrageenan solution was made from Lambda Carrageenan (Sigma-Aldrich) mixed with saline shortly before injection.

The CIA model (n = 4, Lewis female rats, Charles River Laboratories) was generated by injecting collagen emulsion subcutaneously at the tail base 3–4 weeks before the PET scan [13]. In brief, bovine type II collagen 2 mg/ml solution (Chondrex) was mixed with Incomplete Freund’s Adjuvant (MP Biomedicals) using a homogenizer in an ice water bath to make an emulsion shortly before injection. In each animal, 200 μg of collagen emulsion was injected subcutaneously at the tail base under general anesthesia with isoflurane. The injected animal was monitored daily for the development of arthritis.

### Arthritis/paw edema rating

The severity of arthritis or paw edema was assessed using a qualitative scoring system (Table 1) [13]. It is based on the edema and erythema in the hind paws of the rats.

### Mocro-PET scans

**Figure 2:**
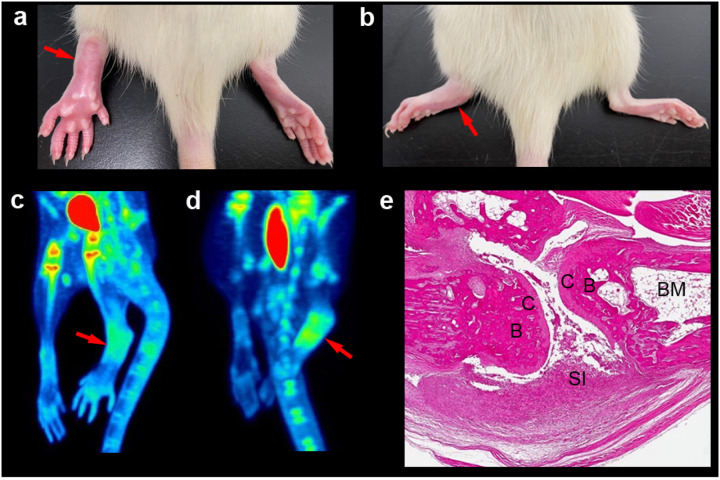
The representative gross findings (**a** and **b**), [^18^F]FGln PET images of the same animals (**c** and **d**), and post-mortem pathology findings (**e**) of carrageenan-induced paw edema (CIPE) and collagen-induced arthritis (CIA). (**a**) Severe erythema and swelling (arrow) were observed in the left hind paw with CIPE compared to the right hind paw. (**b**) Severe erythema and swelling (arrow) were observed in the left hind paw with CIA compared to the right hind paw. (**c**) [^18^F]FGln uptake in the injected hind paw (arrow) was higher compared to the non-injected hind paw by 83%. (**d**) [^18^F]FGln uptake in the hind paw with severe inflammation (arrow) was higher compared to the averaged controls by 173%. In contrast, the contralateral hind paw without inflammation did not show increased [^18^F]FGln uptake. [^18^F]FGln PET images (**c** and **d**) are presented as maximum intensity projection images averaged from 10 to 40 minutes. The organs in red are urinary bladders filled with radioactive urine. (**e**) Hematoxylin & Eosin staining of the hind paw with severe inflammation in a rat with CIA. Profound synovial inflammation is observed in the joint. B=bone; BM=bone marrow; C=cartilage; SI=synovial inflammation.

After a transmission CT scan, 15.7 ± 4.9 MBq of [^18^F]FGln was intravenously injected via the tail vein, followed by a dynamic micro-PET scan for 90 minutes with the Siemens Inveon integrated trimodal imaging system under general anesthesia with isoflurane. In each [^18^F]FGln micro-PET image, a 3-dimensional spherical volume of interest (VOI) was placed in the ankle joint of each paw, based on the anatomical CT image using PMOD software v 4.2 (PMOD Technologies Ltd., Zurich, Switzerland). The VOIs had equal volumes between the right and left sides and did not include any part of the bone, which tends to be biased with radiometabolite accumulation. The standard uptake value in a pixel was calculated as tissue radioactivity concentration from the calibrated PET image divided by injected radioactivity/body weight. The maximum intensity projection images averaged from 10 to 40 minutes were used for visual comparison between conditions (presented in Fig. 2c and d). For quantitative comparisons, the Area Under the Curve (AUC) from 0 to 90 minutes was calculated in the [^18^F]FGln time-activity curve from each VOI. The injected left hind paw of each animal was compared to the non-injected right hind paw of the same animal in the CIPE models. Four non-injected right hind paws of the CIPE models served as controls of the CIA models for comparison of the [^18^F]FGln uptake.

### Statistical analysis

The correlation between the %increase of AUC in the [^18^F]FGln time-activity curve and inflammation severity score in a total of eight hind paws of four CIA rats was analyzed in IBM SPSS v.29 with a non-parametric partial correlation method controlling for subjects (the degree of freedom = 5).

### Postmortem exam

After obtaining micro-PET scans, the animals with CIA were sacrificed, and the paws were amputated and sent for tissue preparation. After decalcification, fixation, sectioning, and Hematoxylin & Eosin (H & E) staining following the published guideline [17], the section slides were digitalized for the visual assessment of inflammation.

## Results

All four rats with the CIPE model developed severe inflammation with erythema and swelling that encompassed the ankle, foot, and digits (score 4 in Table 1) in the carrageenan-injected left hind paws in three hours from the injection (Table 2 and Fig. 2a). Among four rats with the CIA model, two developed severe inflammation (score 4) in both hind paws, one developed asymmetric inflammation with severe (score 4) and mild (score 2) inflammation in each hind paw, and the other one developed severe inflammation in only one hind paw (score 4 and score 0, presented in Fig. 2b) in 3–4 weeks from the injection of the type II collagen emulsion (Table 2).

On the micro-PET scans of the CIPE models, [^18^F]FGln uptake was higher in the injected hind paw with severe inflammation compared to the non-injected hind paw of the same animal by 52–83% (Table 2 and Fig. 2c). In the CIA models, [^18^F]FGln uptake was higher in the hind paw with severe inflammation (score 4) compared to the averaged controls by 54–173% (Table 2 and Fig. 2d). In contrast, [^18^F]FGln uptake in the paw with mild inflammation (score 2) and no inflammation (score 0) of the CIA models was slightly higher (33%) and lower (−7%), respectively, compared to the averaged controls (Table 2). Combined overall, the [^18^F]FGln uptake and inflammation severity score in a total of eight hind paws of four CIA rats showed a significant positive correlation (*r* = 0.88, *P* = 0.009).

The post-mortem pathological exams of the rats with the CIA model confirmed profound inflammation in the paws with severe arthritis (Fig. 2e). Specifically, H & E staining of the section showed typical synovial joint inflammation characterized by inflammatory cell infiltrates, increase in synoviocytes, thickening of synovial lining and sub-lining, and invasion of pannus tissue, as previously described in the CIA model [17].

## Discussion

In our study, [^18^F]FGln uptake was increased in both acute and chronic inflammatory conditions in the rat arthritis and paw edema models. Particularly, in rats with the CIA models, the [^18^F]FGln uptake level showed a significant positive correlation with the severity of induced inflammation despite the small sample size. Our findings suggest the utility of [^18^F]FGln as a potential metabolic imaging marker for inflammatory conditions.

The involvement of glutamine metabolism in the development of joint inflammation has been consistently reported in previous studies with rodent CIA models or human RA cases. For instance, inhibition of the system A family of amino acids transporters, which has glutamine as one of the main substrates, was shown to reduce the development of CIA in rats [18]. The results were mainly attributed to glutamine deprivation with subsequent decrease of circulating monocytes and neutrophils. In addition, glutaminase 1, the metabolizing enzyme from glutamine to glutamate, was shown to be increased in cultured synoviocytes harvested from synovial tissue of RA patients, and deprivation of glutamine led to suppression of the cell proliferation [19]. Another study showed that glutamate is increased in the synovial fluid of RA patients, and the level was positively correlated to the proinflammatory cytokine levels [20]. Thus, the metabolic status of synoviocytes and other inflammatory cells in RA shows significant shifting from normal pathways of aerobic glycolysis to up-regulation of glutaminolysis, which is quite similar to that of the Warburg effect in cancer cells [6]. Indeed, substantial evidence indicates that the metabolic characteristics of synoviocytes in RA have similarities to those of cancer cells [21].

In addition to the synoviocytes in RA, extensive study findings have indicated that major inflammatory cells use glutamine as a dominant energy source in active inflammatory conditions. In previous studies, T-lymphocytes were shown to be dependent on glutamine metabolism in mice models with genetic arthritis or experimental autoimmune encephalomyelitis [22–23]. Glutamine metabolism is also critical in macrophages, particularly for the modulation of the polarization between pro-inflammatory (M1) and anti-inflammatory (M2) phases [24–25]. A recent study with [^18^F]FGln in mice also presented the co-localization of [^18^F]FGln uptake with glutamine transporter-positive macrophages in inflamed atherosclerotic lesions [26]. Neutrophils are also heavily dependent on glutamine to meet their energy demands at inflamed sites where nutrients may be limited [27]. Of note, neutrophils are the most abundant cells present in synovial fluid in patients with RA and have pivotal roles in synovial tissue destruction [28]. Both neutrophils and macrophages also contribute to the hyperacute local inflammation induced by carrageenan in the CIPE model [7–9]. Based on this extensive evidence on the essential roles of glutamine in the metabolic status of inflammatory cells, immunosuppressant drugs, such as methotrexate, leflunomide, and tofacitinib, have been developed and are clinically used for the treatment of RA or inflammatory myositis, with mechanisms to reduce the pro-inflammatory activity of immune cells via modification of metabolic pathways [21].

## Conclusion

Glutamine metabolism is not only essential in cancer cells but also in major inflammatory cells with correlations to activity levels. Our preclinical findings in rat models with arthritis and paw edema suggest the potential utility of [^18^F]FGln PET as a novel metabolic imaging marker in various inflammatory conditions such as inflammatory arthritis and myositis.

## Figures and Tables

**Table 1:** A qualitative scoring system used to assess the severity of paw inflammation [13]

Score	Condition
0	No evidence of erythema and swelling
1	Erythema and mild swelling confined to the tarsals or ankle joint
2	Erythema and mild swelling extending from the ankle to the tarsals
3	Erythema and moderate swelling extending from the ankle to metatarsal joints
4	Erythema and severe swelling encompass the ankle, foot, and digits, or ankylosis of the limb

**Table 2:** Summary of [^18^F]FGln micro-PET findings and the severity of inflammation

Model		%increase of AUC in the [^18^F]FGln time-activity curve^a^	Inflammation severity score
CIPE model			
Rat 1	Left, injected	52	4
	Right	-	0
Rat 2	Left, injected	74	4
	Right	-	0
Rat 3	Left, injected	61	4
	Right	-	0
Rat 4	Left, injected	83	4
	Right	-	0
CIA model*			
Rat 1	Left	173	4
	Right	−7	0
Rat 2	Left	69	4
	Right	33	2
Rat 3	Left	54	4
	Right	75	4
Rat 4	Left	69	4
	Right	60	4

a For the CIPE model, the injected left side was compared to the non-injected right side in each animal. For the CIA model, each side was compared to the average value of the non-injected right side of the CIPE model rats (n = 4).

* %increase of AUC in the [^18^F]FGln time-activity curve showed a significant positive correlation with the inflammation severity score (*r* = 0.88, *P* = 0.009).

CIPE = Carrageenan-Induced Paw Edema; CIA = Collagen-Induced Arthritis; AUC = Area Under the Curve.

## Data Availability

Data related to this study are available from the corresponding authors on reasonable request.
